# Effectiveness and safety of Qixuekang Oral Liquid on vascular health

**DOI:** 10.1515/jtim-2024-0036

**Published:** 2025-01-10

**Authors:** Shantong Jiang, Hongyan Shi, Yanqing Hu, Ning Zhang, Hongyu Wang

**Affiliations:** Department of Vascular Medicine, Peking University Shougang Hospital, Beijing 100144, China; Vascular Health Research Center of Peking University Health Science Center (VHRC-PKUHSC), Beijing 100191, China; Intelligent Heart and Vascular Health Digital Management Research Center of National Institute of Health Data Science at Peking University, Beijing, China; Laoshan Community Health Service Center of Shijingshan District, Beijing 100049, China; Yunnan Baiyao Group Co., Ltd., Kunming 650504, Yunnan Province, China; Heart and Vascular Health Research Center of Peking University Clinical Research Institute (HVHRC-PUCRI), Beijing, China

## To the editor

Vascular diseases, including acute myocardial infarction, stroke, renal vascular disease, and peripheral arterial disease, significantly contribute to mortality and disability rates, particularly in China, where over 3 million deaths annually are attributed to cardiovascular and cerebrovascular diseases. Among survivors, 75% experience varying degrees of disability.^[1]^ Coronary heart disease (CHD) involves multiple risk factors, and its pathophysiological processes are closely related to the body’s immune system.^[2]^ Western medicine, with its single therapeutic target, struggles to comprehensively intervene in the disease. In contrast, Traditional Chinese Medicine (TCM) offers a holistic approach to treating CHD, particularly through pattern differentiation, which tailors treatments to individual syndromes.^[3]^ In TCM, CHD is classified into different syndromes for tailored treatment, with Qi deficiency and blood stasis (QDBS) being one of the common syndromes. The primary treatment approach focuses on promoting blood circulation and removing blood stasis.^[4]^ The Qixuekang Oral Liquid, developed by Yunnan Baiyao Group Co., Ltd. (Approval No. Z53020831) is an innovative product derived from traditional blood-tonifying prescriptions. It is formulated with fresh *San Qi* (Panax notoginseng), *Huang Qi* (Astragalus), *Ren Shen* (Ginseng), and *Ge Gen* (Pueraria Lobata), which can enhance immunity, improve heart function, and alleviate symptoms associated with QDBS.^[5]^

**Figure 1:**
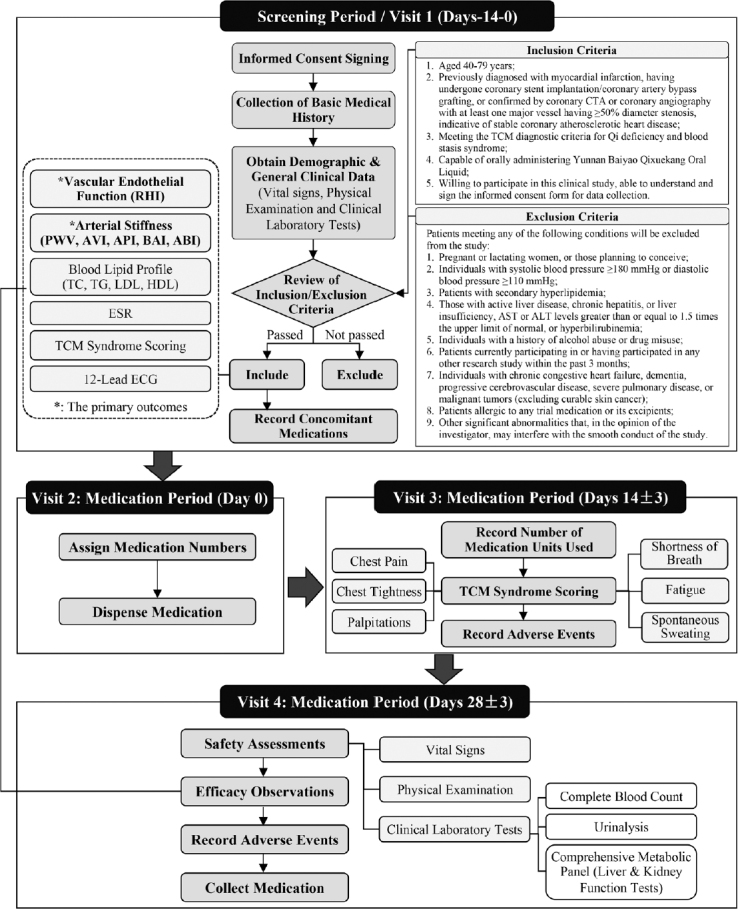
Study flowchart. RHI: reactive hyperemia index; PWV: pulse wave velocity; API: arterial pressure volume index; AVI: arterial velocity pulse index; BAI: brachial-ankle index; ABI: ankle-brachial index; TC: total cholesterol; TG: triglycerides; HDL: high-density lipoprotein cholesterol; LDL: low-density lipoprotein cholesterol; ESR: erythrocyte sedimentation rate; TCM: Traditional Chinese Medicine; ECG: electrocardiogram.

This study is a subgroup of China Digital Heart and Vascular Health Study (CDHVHS, ChiCTR2200062543). This open, prospective, single-arm clinical trial was conducted over a 4-week treatment observation period. The study aimed to evaluate the efficacy and safety of Qixuekang Oral Liquid in patients diagnosed with stable CHD. All participants received 30 mL of Qixuekang Oral Liquid twice daily for four consecutive weeks. The study flowchart was shown in Figure 1.

Data were analyzed using SPSS version 20.0. Results are presented as mean ± standard deviation. Comparisons before and after treatment were conducted using paired sample *t*-tests. A *P*-value of < 0.05 was considered statistically significant.

A total of 109 participants completed the study from December 2023 to April 2024. The study cohort consisted of 75 males and 34 females, with an average age of 67.21 ± 6.49 years (age range 48–79 years). Results showed that after four weeks of continuous Qixuekang Oral Liquid administration in patients with stable CHD, the mean systolic blood pressure decreased from 141.5 mmHg to 134.7 mmHg (*P* < 0.001), and the diastolic blood pressure also saw a significant drop from 79.1 mmHg to 76.1 mmHg (*P* = 0.014). After two weeks of treatment, the TCM syndrome scores showed a substantial reduction from baseline, with scores decreasing from 12.5 to 3.76 (*P* < 0.001). This improvement continued through the four-week period, with the scores at 3.91 at 28 days. The consistent reduction in TCM syndrome scores indicates a rapid and sustained relief of symptoms such as chest pain, shortness of breath, fatigue, and palpitations, which are commonly associated with CHD. In terms of vascular endothelial function, Qixuekang Oral Liquid significantly increased the Reactive Hyperemia Index (RHI) in patients with stable CHD, indicating an improvement in vascular endothelial function. The RHI is a commonly used indicator for assessing vascular endothelial function. It is recognized for its reliable predictive value for high-risk cardiovascular events.^[6,7]^ An RHI value of 1.67 is considered a critical threshold for identifying endothelial dysfunction, with higher RHI values indicating better endothelial function.^[6]^ In our study, after the treatment, the RHI improved from 1.62 to 1.89 (*P* < 0.001), highlighting the efficacy of Qixuekang Oral Liquid in enhancing endothelial function, which is crucial for cardiovascular health and reducing the risk of atherosclerosis and related complications. However, the lack of significant changes in arterial stiffness and other vascular indices such as pulse wave velocity, arterial velocity pulse Index, ankle-brachial index, arterial pressure volume index suggests that Qixuekang Oral Liquid’s primary benefit may be focused on improving endothelial function rather than directly altering arterial stiffness or structural properties. This could imply that while the treatment enhances the dynamic function of the endothelium, it does not significantly impact the more rigid structural aspects of the arteries over the short term. Furthermore, the lack of significant changes in blood lipid levels and ESR supports the conclusion that Qixuekang Oral Liquid’s primary benefits lie in improving endothelial function rather than altering lipid profiles or systemic inflammation. And There were no significant changes in liver function (alanine transaminase, aspartate transaminase, total bilirubin, direct bilirubin, gamma-glutamyl transferase, alkaline Phosphatase), kidney function (blood urea nitrogen, creatinine), or hematological parameters (white blood cells, red blood cells, hemoglobin, platelets, neutrophils, lymphocytes, neutrophil ratio, lymphocyte ratio)(Supplementary Table 1).

In conclusion, Qixuekang Oral Liquid demonstrates significant potential as a safe and effective therapeutic option for enhancing vascular health in patients with stable CHD. The study reveals notable improvements in endothelial function, as indicated by increased RHI values, and a statistically significant reduction in both systolic and diastolic blood pressures after four weeks of treatment. While Qixuekang Oral Liquid did not significantly impact arterial stiffness or lipid levels, it showed no adverse effects on liver or kidney functions, supporting its role as a complementary therapy in managing CHD. These findings underscore the importance of integrating TCM with conventional treatments to optimize cardiovascular care. Further research is warranted to elucidate the specific mechanisms of action and long-term cardiovascular outcomes associated with Qixuekang Oral Liquid, laying the groundwork for future studies in this area.

### Supplementary Information

Supplementary materials are only available at the official site of the journal (www.intern-med.com).

## Supplementary Material

Supplementary Material
