# Supplementary material for: Effectiveness and safety of Qixuekang Oral Liquid on vascular health
Source: J Transl Int Med. 2025 Jan 10;12(6):618–20. doi: 10.1515/jtim-2024-0036 (PMC11720926; doi:10.1515/jtim-2024-0036)
Supplement: Supplementary file 1 — Supplementary Material [file jtim-2024-0036_sm.pdf]

Supplementary Table 1: Results of the efficacy and safety of Qixuekang Oral Liquid in the patients with stable coronary heart disease

Characteristics	Sample size	Before treatment (mean ± SD)	After treatment (mean ± SD)	*P* value
SBP, mmHg	109	140.7 ± 14.9	134.7 ± 16.4	< 0.001
DBP, mmHg	109	78.9 ± 11.2	76.1 ± 12.3	0.014
RHI	109	1.62 ± 0.32	1.89 ± 0.35	< 0.001
Right PWV, cm/s	109	1642.23 ± 326.57	1648.32 ± 270.50	0.812
Left PWV, cm/s	109	1558.94 ± 250.78	1535.15 ± 259.78	0.207
AVI	109	24.90 ± 8.88	23.36 ± 8.04	0.77
API	109	32.93 ± 7.21	32.17 ± 7.53	0.284
Right BAI	109	0.97 ± 0.09	0.98 ± 0.11	0.208
Left BAI	109	0.97 ± 0.13	0.97 ± 0.11	0.763
Right ABI	109	0.99 ± 0.11	0.98 ± 0.12	0.360
Left ABI	109	0.97 ± 0.11	0.96 ± 0.11	0.248
TCM Syndrome Score	109	12.5 ± 3.4	3.76 ± 2.80	< 0.001*
			3.91 ± 2.60	0.606#
TC, mmol/L	109	3.96 ± 1.01	4.01 ± 1.10	0.258
TG, mmol/L	109	1.69 ± 0.91	1.57 ± 1.01	0.141
HDL, mmol/L	109	1.19 ± 0.26	1.21 ± 0.25	0.153
LDL, mmol/L	109	2.56 ± 0.80	2.67 ± 0.76	0.190
ESR, mm/h	109	9.43 ± 6.21	9.42 ± 6.33	0.983
ALT, *U*/L	109	22.14 ± 10.43	22.33 ± 11.81	0.898
AST, *U*/L	109	26.63 ± 12.85	24.17 ± 10.71	0.060
Tbil, μmol/L	109	16.88 ± 7.03	15.75 ± 6.12	0.161
Dbil, μmol/L	109	5.34 ± 2.31	5.01 ± 2.27	0.239
γ-GT, *U*/L	109	26.57 ± 13.06	27.71 ±15.74	0.374
ALP, *U*/L	109	61.02 ± 14.11	67.04 ± 25.74	0.006
BUN, μmol/L	108	5.71 ± 1.60	5.96 ± 1.85	0.223

Cr, μmol/L	108	72.38 ± 18.05	74.95 ± 17.99	0.190
WBC, 10^9/L	107	6.00 ± 1.50	5.80 ± 1.37	0.056
RBC, 10^{12}/L	107	4.57 ± 0.51	4.57 ± 0.50	0.929
HGB, g/L	106	140.27 ± 13.95	141.42 ± 17.37	0.334
PLT, 10^9/L	106	192.57 ± 42.18	194.15 ± 43.36	0.599
NEU, 10^9/L	109	3.54 ± 1.16	3.40 ± 0.99	0.125
LYM, 10^9/L	109	1.89 ± 0.57	1.84 ± 0.57	0.195
GR%, %	109	58.13 ± 8.16	58.07 ± 8.07	0.939
LY%, %	109	32.09 ± 8.57	32.11 ± 8.24	0.977

*Comparison between scores before treatment and at 14 days; #Comparison between scores at 14 days and at 28 days. SBP: Systolic Blood Pressure; DBP: Diastolic Blood Pressure; RHI: reactive hyperemia index; PWV: pulse wave velocity; API: arterial pressure volume index; AVI: arterial velocity pulse index; BAI: brachial-ankle index; ABI: ankle-brachial index; TC: total cholesterol; TG: triglycerides; HDL: high-density lipoprotein cholesterol; LDL: low-density lipoprotein cholesterol; ESR: erythrocyte sedimentation rate; ALT: Alanine Transaminase; AST: Aspartate Transaminase; Tbil: Total bilirubin; Dbil: Direct bilirubin; γ-GT: gamma-glutamyltransferase; ALP: Alkaline Phosphatase; BUN: blood urea nitrogen; Cr: creatinine; WBC: white blood cells; RBC: red blood cells; HGB: hemoglobin; PLT: platelets; NEU: neutrophils; LYM: lymphocytes; GR%: neutrophil ratio; LY%: lymphocyte ratio.
